# UAV Sensor Fault Detection Using a Classifier without Negative Samples: A Local Density Regulated Optimization Algorithm†

**DOI:** 10.3390/s19040771

**Published:** 2019-02-13

**Authors:** Kai Guo, Liansheng Liu, Shuhui Shi, Datong Liu, Xiyuan Peng

**Affiliations:** School of Electronics and Information Engineering, Harbin Institute of Technology, Harbin 150080, China; guok@hit.edu.cn (K.G.); lianshengliu@hit.edu.cn (L.L.); shishuhui@hit.edu.cn (S.S.)

**Keywords:** fault detection, sensors, unmanned aerial vehicles, flight control system, one-class support vector machine, local density

## Abstract

Fault detection for sensors of unmanned aerial vehicles is essential for ensuring flight security, in which the flight control system conducts real-time control for the vehicles relying on the sensing information from sensors, and erroneous sensor data will lead to false flight control commands, causing undesirable consequences. However, because of the scarcity of faulty instances, it still remains a challenging issue for flight sensor fault detection. The one-class support vector machine approach is a favorable classifier without negative samples, however, it is sensitive to outliers that deviate from the center and lacks a mechanism for coping with them. The compactness of its decision boundary is influenced, leading to the degradation of detection rate. To deal with this issue, an optimized one-class support vector machine approach regulated by local density is proposed in this paper, which regulates the tolerance extents of its decision boundary to the outliers according to their extent of abnormality indicated by their local densities. The application scope of the local density theory is narrowed to keep the internal instances unchanged and a rule for assigning the outliers continuous density coefficients is raised. Simulation results on a real flight control system model have proved its effectiveness and superiority.

## 1. Introduction

Unmanned aerial vehicles (UAVs) are drawing significantly increasing attention in recent years due to their convenience and flexibility in executing various tasks compared to manned aircrafts, however, the accident rate of UAVs is much higher than that of manned aircraft because of the immature equipment technology and the lack of effective on-board system health management and diagnostic technology [1,2]. Due to the high altitude and long flight time requirements of UAVs, their airborne equipment must be as light as possible to load more fuel, which restricts the complexity of the UAV diagnosis systems. In addition, the extension of flight time also increases the possibility of failure. Therefore, there is a critical requirement for the application of effective fault detection approaches.

The flight control system, which is responsible for the accomplishment of flight tasks, is the core of the UAV. Through comparing the actual state of the UAV indicated by the sensor data with the desired state, it sends commands to actuators to make the actual state follow the desired state, completing the closed-loop control process [3,4]. Sensors provide sensing data for the flight control system to make analysis and generate control commands, and faulty sensor data will cause erroneous commands and undesirable consequences. Also, flight sensors are often exposed to the rapidly changing pressure and temperature of the air, increasing its possibility of breakdown. Therefore, fault detection for UAV sensors is quite essential for ensuring flight security.

Many fault detection approaches for sensors have been developed in recent years, which are mainly divided into two categories: model-based approaches and data-driven approaches [5,6]. Model-based approaches can achieve high detection performance and meanwhile provide detailed interpretable fault information under the condition that accurate physical model of the monitored system can be obtained [7,8,9]. However, accurate physical models for UAVs are quite difficult to set up, which has limited their application [10,11]. In comparison to model-based approaches, data-driven methods do not require recognition of the complex physical mechanism, and take full advantage of the information contained in sensor data [12,13]. Faults can be accurately detected by various data-driven approaches based on the variation in data characteristics [14,15,16].

Data-driven fault detection methods are mainly divided into three different classes: supervised detection approaches, semi-supervised detection approaches and unsupervised detection approaches, as shown by Wang et al. [17], Zhang et al. [18] and Zhang et al. [19]. In the supervised case, the training dataset contains both normal and faulty instances, and the detection model is applied on the test dataset for differentiating the normal from the faulty as illustrated by Yuan et al. [20]. In the semi-supervised situation, the approach only models normal instances and the instances that do not comply with the established model are labeled as faults as shown by Kittler et al. [21]. For unsupervised detection method, a boundary enclosing all possible normal data points is set up, which is utilized as a decision principle for judging the property of a test point shown by Jiang et al. [22]. As for the UAV, faulty instances are difficult to acquire and limited in amount, and often without label information [23,24]. Therefore, unsupervised fault detection techniques appeal more attractive for conducting fault detection of UAV sensors.

Three categories of unsupervised fault detection algorithms have been developed for dealing with the challenge of only using intrinsic information about normal data as shown by Liu et al. [25]: (i) *Density-based methods*. Density-based methods measure the density of the instances and treat low-density area points as faults, like one-class Gaussian mixture model (OCGMM) approach as shown by Ilonen et al. [26] and local outlier factor (LOF) approach proposed by Song et al. [27]. (ii) *Reconstruction-based methods*. Reconstruction-based methods assume that the points are dense enough and the instances deviating from the reconstructed model will be regarded as faulty, like K-means given by Capozzoli et al. [28] and principle component analysis (PCA) given by Zhou et al. [29]. (iii) *Boundary-based methods*. Boundary-based methods, such as one-class support vector machine (OCSVM) presented by Yan et al. [30], are capable of establishing a tight and smooth boundary enclosing normal data with kernel tricks, and can cope with complex nonlinear dataset. Compared to the two former categories, OCSVM has better performance in detecting sensor faults with small amplitude in nonlinear conditions by establishing a close enclosure of the instances. Also, it can monitor multi parameters simultaneously with well-established features and the correlation between the flight parameters of the UAV can be well considered.

In UAV sensor data, there often exist outliers that deviate from the majority of the data, which have significant influence on the decision boundary of OCSVM [31,32]. To maintain high detection accuracy in UAV sensor fault detection when there exist outliers, it requires a special mechanism for dealing with them, which is not provided by OCSVM itself. A robust one-class SVM approach was proposed to solve the problem of the influence caused by outliers in fault detection by Xiao et al. [33]. The probable outliers were identified and removed so that the decision boundary would enclose the core of the cluster. The method was validated on online public dataset. However, the outliers were directly removed and a hard classification of the instances was unavoidable in the preprocessing stage. The performance of OCSVM was subjected to the accuracy of judging outliers. The proposed algorithm shared the common defect of sequential model fusion that the accuracy of the latter algorithm is always limited by the former one. Two approaches were raised to make OCSVM robust to outliers in the training data by taking the effects of outliers into consideration while establishing the decision boundary by Amer et al. [34]. The outliers were assigned different coefficients during the training process to decrease their effects and the approaches were validated on UCI datasets. However, the original structure of OCSVM was largely changed in both the two methods, and all the instances were allocated new coefficients. The performance improvement of the former approach was not quite obvious, and the latter one required a prejudgment of the outliers to accomplish its training process. Therefore, dealing with the outliers in UAV sensor fault detection is still a challenging issue.

In this paper, an optimized OCSVM approach regulated by local density is proposed for UAV sensor fault detection. By selecting and constructing appropriate features, the proposed approach can achieve high detection performance for UAV sensor fault. The training dataset is first divided into internals and externals according to their distances to the center and the application scope of the density theory is narrowed to keep the internals unchanged. Then the tolerance coefficients of the externals are reassigned according to their extent of abnormality and a rule is raised for measuring it based on their local densities. The influence of the outliers on the monitoring model is thus mitigated and the principle structure of OCSVM is reserved. The proposed approach can achieve robust performance to outliers without requiring priori knowledge of the instances and do not need to make a prejudgment of the outliers before the training process. Comparison experiments with several representative unsupervised fault detection approaches are conducted on UAV sensor data to prove its effectiveness.

The rest of this paper is organized as follows: the theorem of the proposed approach is illustrated in details in Section 2. In addition, the motivation for improving OCSVM is illustrated and a rule for regulating the tolerance coefficients of the outliers is raised. A framework for detecting UAV sensor faults using the proposed approach is also given. Section 3 presents comparison experiments on UAV sensor data and two different types of sensor fault are considered. The results have proved the performance of the proposed approach. Conclusions and future works are discussed in Section 4.

## 2. UAV Sensor Fault Detection with Optimized Classifier Regulated by Local Density

In this section, the influence of outliers on the performance of the OCSVM approach in detecting UAV sensor faults is first illustrated. The theorem of the proposed optimized classifier to mitigate the influence of the outliers on the decision boundary is then presented. In addition, a rule for regulating the tolerance coefficients of the instances based on local density theory is proposed. The application scope of the local density theory is further narrowed to avoid useless computation and keep the internal points unchanged. Finally, a framework for detecting UAV sensor faults using the proposed approach is proposed.

### 2.1. Problem Statement

**Figure 1:**
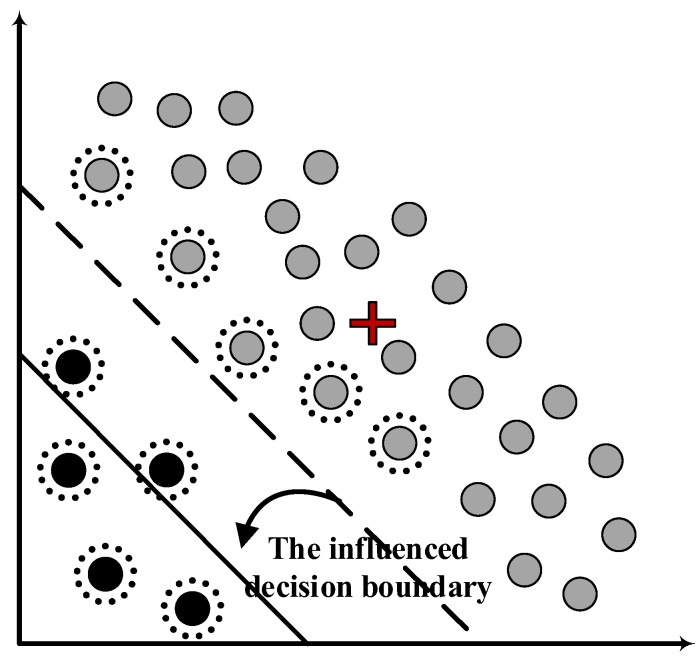
The illustration for the influence of outliers on the decision boundary of OCSVM.

In UAV sensor data, there often exist outliers that deviate from the majority of the data, which have significant influence on the decision boundary of OCSVM. As shown in Figure 1, the original decision boundary of OCSVM is represented by the dashed line and the grey circles above surrounded by dash lines are the corresponding support vectors of the decision model. After the outliers represented by the black circles near the origin are mixed into the training dataset, they become new support vectors and the new decision boundary represented by the solid line has been shifted towards the origin, leading to the degradation in detection accuracy.

The common approach for dealing with this issue is to identify and eliminate the outliers before training the OCSVM, however, this requires an additional preprocessing stage to label them as normal or abnormal. The problem is that the preprocessing stage also depends on a particular anomaly detection approach for labeling abnormal instances, and it seems not reasonable to apply OCSVM based on the former because its accuracy is limited by the former approach. Therefore, dealing with the outliers still remains a challenging issue.

### 2.2. Optimized Classifier without Negative Samples

Given the training dataset $X={\left\{x_{i}\right\}}_{i=1}^{n},x_{i}\in R^{m}$, a nonlinear mapping ϕ from the original space $R^{m}$ to a high dimensional feature space χ is implicitly defined for dealing with nonlinear characteristics of the dataset by a kernel function:

(1) $$K\left(x_{i},x_{j}\right)=\left(\phi \left(x_{i}\right)\cdot \phi \left(x_{j}\right)\right)$$

such as the Gaussian kernel $K\left(x_{i},x_{j}\right)=e^{-{\left\|x_{i}-x_{j}\right\|}^{2}/\gamma }$, where γ is the coefficient for modulating the performance of the Gaussian kernel. The purpose of basic OCSVM is to establish a compact hyperplane:

(2) w⋅ϕ(x)−ρ=0

which encloses the training instances tightly in χ to linearly separate the mapped samples $\phi \left(x_{i}\right),i=1,\ldots ,n$ from the origin with maximum Euclidean distance ρ/‖w‖. w is the coefficient vector of the decision hyperplane, and ρ is the intercept of the hyperplane. To solve the problem of hard classification, a slack factor $\xi_{i}\geq 0$ is introduced to each sample point so that the training samples are allowed to fall on the other side of the classification boundary. The optimization problem for basic OCSVM described above can be formulated as follows:

(3) $$\begin{array}{l} \underset{w,\xi ,\rho }{\min}\left(\frac{1}{2}{\left\|w\right\|}^{2}+\frac{1}{vn}\sum_{i=1}^{n}\xi_{i}-\rho \right)\\ s.t.\;\left(w\cdot \phi \left(x_{i}\right)\right)\geq \rho -\xi_{i}\;\mathrm{and}\;\xi_{i}\geq 0\;\forall i \end{array}$$

where v∈(0,1] is a predefined percentage parameter for controlling the performance of basic OCSVM that represents the upper bound on the fraction of outliers and the lower bound on the fraction of support vectors simultaneously [35]. 

In order to mitigate the influence of the outliers on the decision boundary, tolerance coefficients $c_{i}$ are integrated into the convex quadratic programming problem of basic OCSVM represented by Equation (3) according to their local densities, and the formulation for the optimized classifier becomes:

(4) $$\begin{array}{l} \underset{w,b,\xi_{i}}{\min}\left(\frac{1}{2}{\left\|w\right\|}^{2}+\frac{1}{vn}\sum_{i=1}^{n}\xi_{i}-\rho \right)\\ s.t.\;\left(w\cdot \phi \left(x_{i}\right)\right)\geq \rho -c_{i}\xi_{i}\;,\xi_{i}\geq 0,\;\forall i \end{array}$$

The theorem of regulating $c_{i}$ for different sample points is briefly illustrated here and details are given in the next section to make the logic clear. $c_{i}$ will be set to 1 for the internal instances to reserve the original optimization problem, and for the externals, they are assigned different values according to their extents of abnormality, i.e., their extents of differences to their neighbors. The instance with significant difference to its neighbors will be assigned a large value and a large $c_{i}$ will allow $x_{i}$ to fall between the origin and the decision boundary with a large distance to the boundary. This will reduce of the influence of the instance on solving the decision boundary. The Lagrange function for the problem represented by Equation (4) is introduced as follows by utilizing multipliers $\alpha_{i},\beta_{i}\geq 0$:

(5) $$L\left(w,\xi ,\rho ,\alpha ,\beta \right)=\frac{1}{2}{\left\|w\right\|}^{2}+\frac{1}{vn}\sum_{i=1}^{n}\xi_{i}-\rho -\sum_{i=1}^{n}\alpha_{i}\left(\left(w\cdot \phi \left(x_{i}\right)\right)-\rho +c_{i}\xi_{i}\right)-\sum_{i=1}^{n}\beta_{i}\xi_{i}$$

By setting the partial derivatives of variables w, $\xi_{i}$ and ρ to zero, i.e., $\frac{\partial L}{\partial w}=\frac{\partial L}{\partial \xi_{i}}=\frac{\partial L}{\partial \rho }=0$, the following formulas can be obtained as:

(6) $$w=\sum_{i=1}^{n}\alpha_{i}\phi \left(x_{i}\right)$$

(7) $$c_{i}\alpha_{i}=\frac{1}{vn}-\beta_{i}$$

(8) $$\sum_{i=1}^{n}\alpha_{i}=1$$

In Equation (6), all the instances $x_{i}\in R^{m},i=1,\ldots ,n$ satisfying $\alpha_{i}>0$ are called support vectors, which lie on the decision boundary. From Equation (7), it can be seen that $c_{i}$ proposed in this paper has direct influence on coefficient $\alpha_{i}$, which determines the final decision model. Together with Equations (1) and (2), the parameter ρ in Equation (5) can be figured out by each support vector mentioned above as:

(9) $$\rho =\left(w\cdot \phi \left(x_{i}\right)\right)=\sum_{j=1}^{n}\alpha_{j}K\left(x_{j},x_{i}\right)$$

By combining Equations (6)–(8) with Equation (5), the dual form of Equation (5) can be expressed as:

(10) $$\begin{array}{l} \underset{\alpha }{\min}\frac{1}{2}\sum_{i=1}^{n}\sum_{j=1}^{n}\alpha_{i}\alpha_{j}K\left(x_{i},x_{j}\right)\\ s.t.\;0\leq \alpha_{i}\leq \frac{1}{c_{i}vn},\sum_{i=1}^{n}\alpha_{i}=1 \end{array}$$

By solving the problem of Equation (10), the coefficients $\alpha_{i},i=1,\ldots ,n$ can be obtained, and the decision boundary can be expressed as:

(11) $$f\left(x\right)=\mathrm{sgn}\left(\sum_{i=1}^{n}\alpha_{i}K\left(x_{i},x\right)-\rho \right)$$

The merits of the proposed approach lie in the following three aspects: (i) By regulating the tolerance coefficients of the instances, the influence of the outliers is mitigated in a soft way. While most existing algorithms require a preprocessing stage to eliminate the outliers or a filtering process, the proposed algorithm is well integrated in the basic level and the judgment of the properties of the outliers is not required. (ii) The proposed method does not demand a priori knowledge of the instances and is applicable with a small amount of training instances available. (iii) The basic structure of OCSVM approach is reserved and therefore its advantages are inherited, and therefore it is computationally efficient because its detection model is just determined by the support vectors.

### 2.3. Rule for Computing the Tolerance Coefficients

For UAV sensor data, the densities of the externals are generally lower than the internals, and the outliers belonging to the externals have much smaller local densities. Therefore, the extent of abnormality of an instance can be well indicated by its local density, and based on this characteristic, a rule for reassigning the instances continuous tolerance coefficients is proposed to mitigate the influence of the outliers.

Because OCSVM mainly focuses on figuring out a tight enclosure for the instances, its boundary will only be determined by the external instances in the dataset and the internals have little effect on the decision boundary. Therefore, the application scope of the local density theory is narrowed to keep the internals unchanged and avoid worthless computation. The local density theory can be utilized specifically to deal with the externals in their neighborhoods, without involving the internals, which deeply fits the idea of OCSVM, modeling all the instances with only the externals.

**Figure 2:**
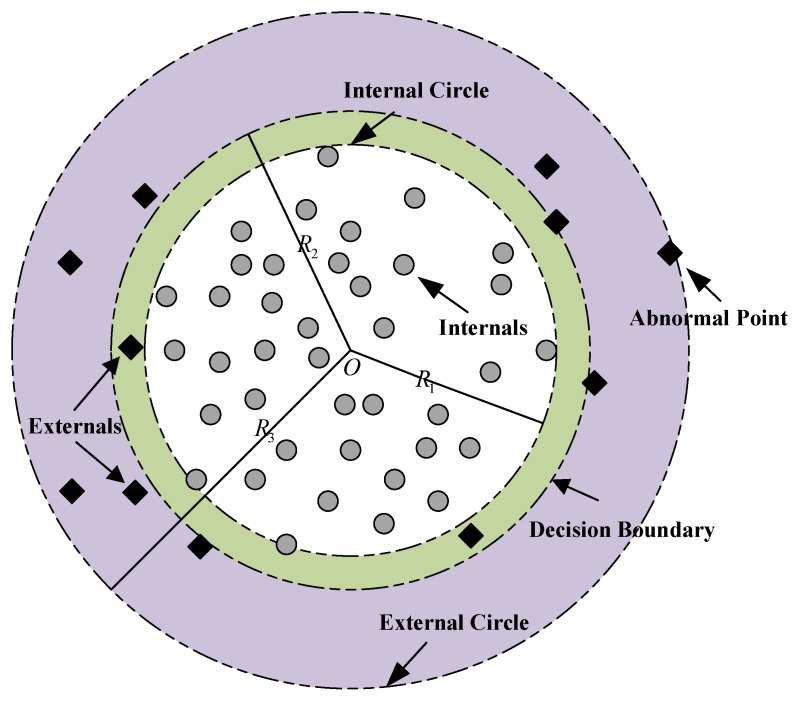
The illustration of selecting instances that require analysis with local density theory.

As shown in Figure 2, the grey circles inside the internal circle with a radius of $R_{1}$ are named the internals and the black squares between the internal circle and the external circle with a radius of $R_{3}$ are named the externals. For the instances shown in Figure 2, the classical OCSVM approach will construct a classification boundary with some externals selected as support vectors, without taking advantage of the internals. Therefore, it is not necessary to apply the local density theory on the internals, which do not contribute to the decision model, and only the externals are focused on in the proposed approach to avoid worthless computation. An important issue that requires attention is how to define the value of $R_{1}$ to control the proportion of the externals. The proportion of externals cannot be accurately defined according to the theory of OCSVM, and thus it is set to a minimum value large enough to contain all the possible instances that may contribute to the decision boundary, so that the maximum amount of samples can be kept unchanged.

In order to compute the tolerance coefficients of the externals, their local densities are first calculated. Assume $x_{1}$, $x_{2}$ and $x_{3}$ are three arbitrary externals in the training dataset $X={\left\{x_{i}\right\}}_{i=1}^{n},x_{i}\in R^{m}$, and k represents the number of neighbors for them. $d\left(x_{1},x_{2}\right)$ denotes the distance between instances $x_{1}$ and $x_{2}$. Then the local density of $x_{1}$ can be figured out by the following four steps:

*Step 1*. Calculating the k-distance of $x_{1}$.

The k-distance of $x_{1}$, denoted as $\text{k-distance}\left(x_{1}\right)$, is equal to $d\left(x_{1},x_{2}\right)$ when the following two conditions are satisfied:

- for at least k instances $x_{i}\in X\backslash \{x_{1}\}$, it holds that $d\left(x_{1},x_{i}\right)\leq d\left(x_{1},x_{2}\right)$, and
- for at most k−1 instances $x_{i}\in X\backslash \{x_{1}\}$, it holds that $d\left(x_{1},x_{i}\right)<d\left(x_{1},x_{2}\right)$.

*Step 2*. Finding the k-distance neighborhood of $x_{1}$.

The k-distance neighborhood of $x_{1}$, denoted as $N_{\text{k-distance}\left(x_{1}\right)}\left(x_{1}\right)$, contains each instance with a distance from $x_{1}$ not greater than its k-distance, i.e.,

(12) $$N_{\text{k-distance}\left(x_{1}\right)}\left(x_{1}\right)=\left\{x_{j}\in X\backslash \left\{x_{1}\right\}|d\left(x_{j},x_{1}\right)\leq \text{k-distance}\left(x_{1}\right)\right\}$$

Later in this paper, $N_{\text{k-distance}\left(x_{1}\right)}\left(x_{1}\right)$ is simplified as $N_{k}\left(x_{1}\right)$ for conciseness.

*Step 3*. Calculating the reachability distance of instance $x_{1}$ w.r.t. instance $x_{3}$.

The reachability distance of instance $x_{1}$ w.r.t. instance $x_{3}$ is defined as

(13) $${\text{reach-dist}}_{k}\left(x_{1},x_{3}\right)=\max\left\{\text{k-distance}\left(x_{3}\right),d\left(x_{1},x_{3}\right)\right\}$$

which indicates that if instance $x_{1}$ is far from instance $x_{3}$, the reachability distance between them is equal to their actual distance. However, if instance $x_{1}$ is close to instance $x_{3}$, the actual distance will be replaced by the k-distance of $x_{3}$ to reduce the fluctuation of ${\text{reach-dist}}_{k}\left(x_{1},x_{3}\right).$

*Step 4*. Measuring the local reachability density of instance $x_{1}$.

In order to measure the local reachability density of instance $x_{1}$, the reachability distances of the instances within the k-distance neighborhood of $x_{1}$ are utilized. Its formulation is defined as

(14) $${\mathrm{lrd}}_{k}\left(x_{1}\right)=1/\left[\frac{\sum_{x_{i}\in N_{k}\left(x_{1}\right)}{\text{reach-dist}}_{k}\left(x_{1},x_{i}\right)}{\left|N_{k}\left(x_{1}\right)\right|}\right]$$

By executing the four steps given above sequentially, we can acquire the local reachability density of each external in the dataset, which equals the inversion of the average reachability distance of $x_{1}$ in its k-distance neighborhood. Before supplementing the local density information of the instances into OCSVM, there still requires a mapping rule for transforming the densities of the instances into tolerance coefficients with reasonable values. The exponential function is adopted here to compute the corresponding coefficients for the externals, which is gently in the initial part and steep in the latter part so that the externals with large extent of abnormality can be severely punished with the normal externals nearly unchanged. The rule for computing the tolerance coefficient of each external based on ${\mathrm{lrd}}_{k}\left(x_{1}\right)$ is:

(15) $$c_{i}=\exp\left(\mu /\left({\mathrm{lrd}}_{k}\left(x_{1}\right)-\eta \right)\right)$$

in which μ and η are coefficients regulating the mapping scale of each external $x_{1}$, and the value of them can be confirmed through optimization. $c_{i}$ is the tolerance coefficient that we need to regulate the tolerance coefficients of the externals. For the internals, their tolerance coefficients are set to 1 to keep the optimization problem unchanged.

### 2.4. Framework for the Proposed UAV Sensor Fault Detection Approach

**Figure 3:**
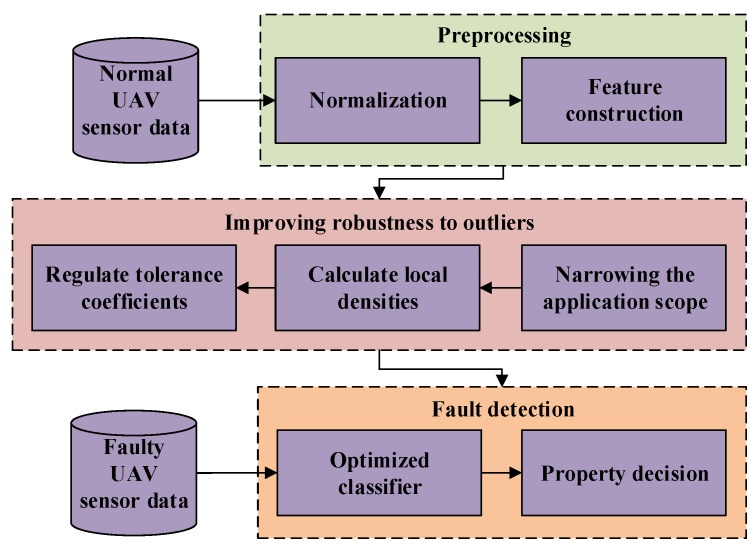
The framework of proposed UAV sensor fault detection approach.

Fault detection for UAV sensors adopting the proposed approach can be accomplished in the following three major stages as shown in Figure 3:

*Stage 1. Preprocessing*.

The sensor data of the UAV are first standardized to eliminate the influence of parameters with large value ranges on the others. Then, the features appropriate for training OCSVM are selected and further constructed by computing the derivatives of the parameters to provide more information of the UAV.

*Stage 2. Improving the robustness of OCSVM to outliers.*

To mitigate the influence of outliers on the decision boundary of OCSVM, the application scope of the local density theory is narrowed and the local densities of the externals are calculated to regulate their tolerance coefficients, so that the robustness of OCSVM to outliers can be improved. An optimized classifier without negative samples is established.

*Stage 3. Fault detection for UAV sensors*.

UAV sensor data with injected faults are tested adopting the optimized classifier to validate its detection performance. The proposed approach can learn the multidimensional space distribution of the features for the UAV through sensor data without negative sample points, and meanwhile does not require large amount of training data, making it superior in UAV sensor fault detection.

## 3. Experiments and Results

In this section, the performance of the proposed approach is compared with several other approaches on UAV sensor data generated from the simulation model of a real UAV under different fault modes. The characteristics of the generated sensor data are illustrated in details. Experimental results are analyzed and discussed.

### 3.1. UAV Data Description

**Figure 4:**
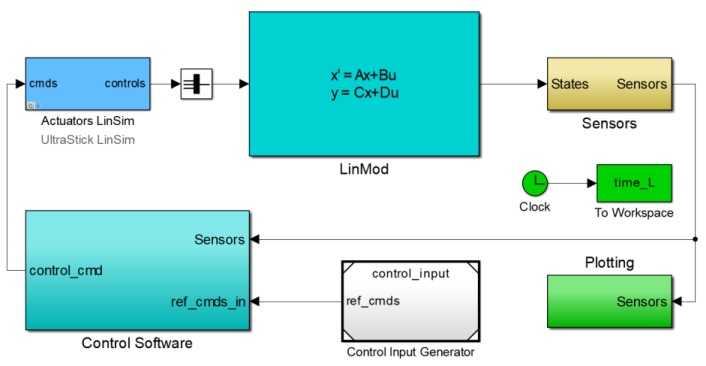
The simulation model of the UAV.

**Figure 5:**
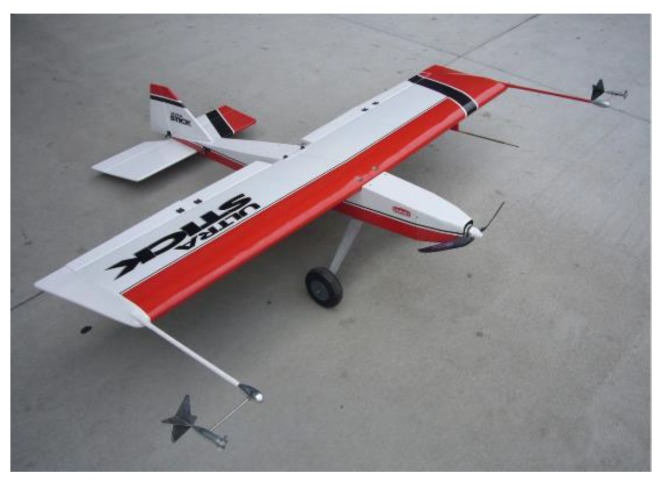
The Ultra Stick 120.

Because real UAV faulty instances are hard to acquire and limited in amount, current research work on UAV fault detection still heavily rely on simulation for validating and comparing the performance of fault detection approaches [36,37,38]. In this paper, for the purpose of verifying the effectiveness of the proposed approach, the UAV simulation model from the UAV lab of University of Minnesota as shown in Figure 4 is adopted for conducting validation experiments, which is established according to a real UAV named Ultra Stick 120 as shown in Figure 5 [39]. The flight control law in the simulation model is used in real flights of the UAV and its effectiveness has been validated [40]. Because real UAV flight data do not contain faulty instances, its simulation model is adopted for evaluating the performance of fault detection approaches by artificially injecting sensor faults into the model. Two common types of sensor faults, constant deviation fault and drift fault, are considered. The outputs of the model under different fault modes are analyzed adopting the proposed approach and other typical unsupervised methods, and the detection results are compared.

There are in total 13 parameters in the simulation model of the UAV flight control system, and their definitions are given in Table 1 for better understanding. The sample time for the simulation model is set to 0.02 s. Generally, the UAV system is decoupled into the longitudinal and the lateral subsystems to execute flight control commands. The two subsystems are equal for validating the performance of the flight control system, therefore, the lateral subsystem is adopted for conducting validation experiments in this paper. The parameters selected for monitoring the status of the lateral subsystem are given in Table 2. Furthermore, the derivatives for them are also constructed and adopted as extra variables because they contain extra fault information, which can be utilized to improve the detection rate of sensor faults.

The UAV is commanded to fly straight and level approximately with all the attitude parameters mentioned in Table 2 around 0. This is a typical flying status of the UAV, which occupies its most flight time. Therefore, it is taken as the fault-free flight condition in this paper. Two typical types of sensor faults are considered in this paper, i.e., constant deviation fault and drift fault. The roll rate p is selected to inject faults to simulate actual faults in the gyroscope, which is an important flight sensor adopted to measure the attitude angles and the attitude angular velocities of the UAV. The advantage of injecting fault on the simulation model of the UAV is that it is not a simple addition on a single parameter, but its influence on the other parameters is also well reflected through the closed-loop control process. The aerodynamic characteristics of the flight control system are considered while establishing the simulation model, which makes the fault injection process more significant.

The faults of the sensor are modeled as follows:

(16) $$y_{m}\left(k\right)=s\left(k\right)y_{c}\left(k\right)+d\left(k\right)$$

where $y_{c}\left(k\right)$ and $y_{m}\left(k\right)$ represent the expected output and the real output of the sensor, respectively. d(k) is the deviation of the output and s(k) is the gain coefficient. Then the constant deviation fault and drift fault mentioned above can be expressed as follows:

- Constant deviation fault. s(k)=1 and d(k) is a constant value.
- Drift fault. s(k)=1 and the value of d(k) increases or decreases gradually along with time.

**Figure 6:**
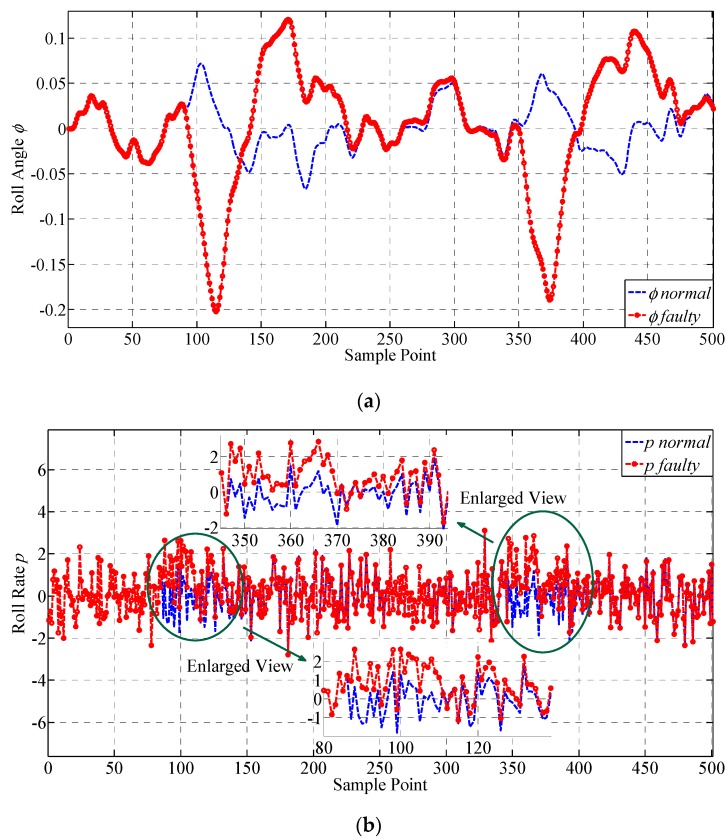

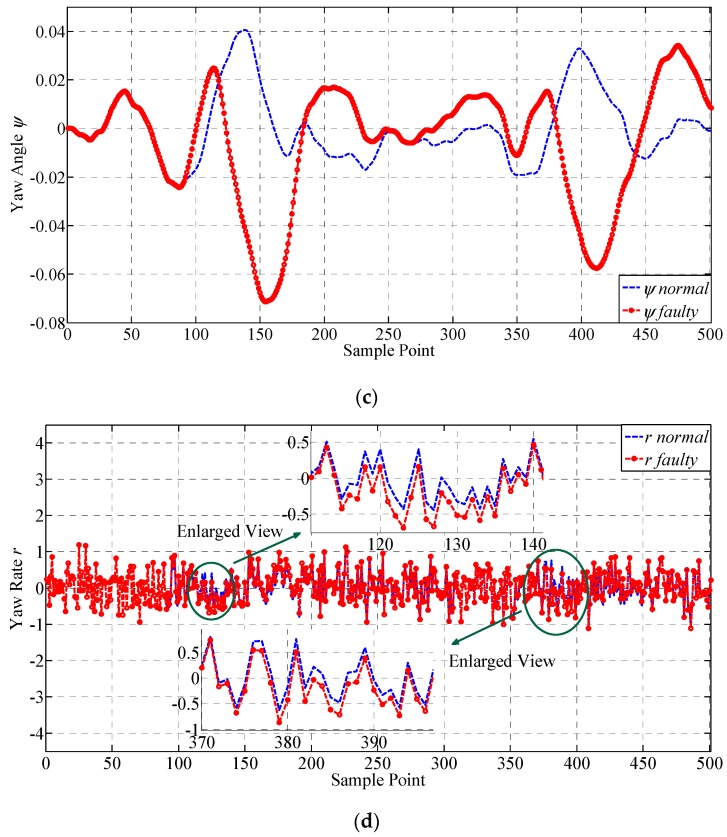
Illustration of the influence of constant deviation fault on p to the lateral system. The curves of lateral outputs before and after injecting constant deviation fault are presented, respectively. (**a**) Roll Angle ϕ; (**b**) Roll Rate p; (**c**) Yaw Angle ψ; (**d**) Yaw Rate r.

**Figure 7:**
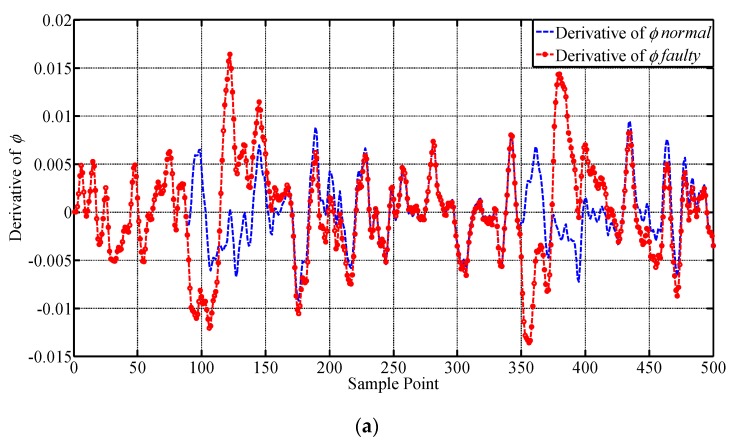

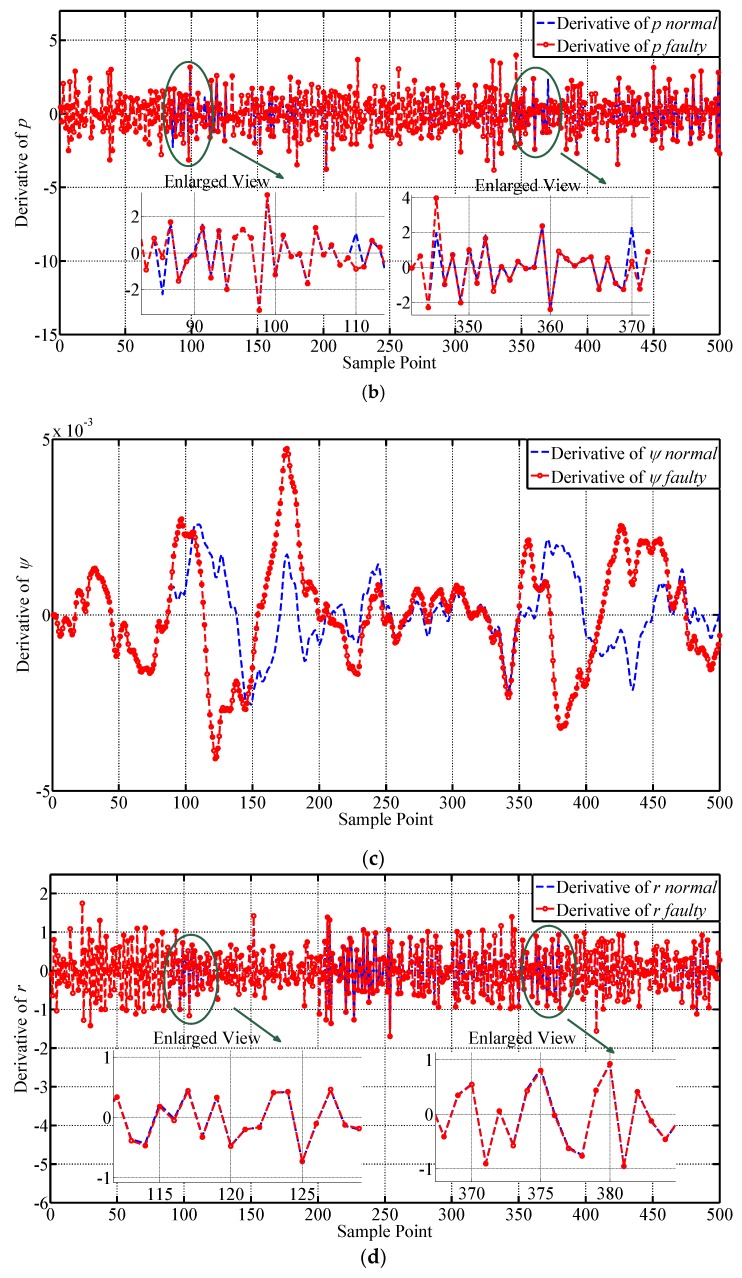
The illustration for the derivatives of the monitored lateral parameters. The curves of the derivatives of the lateral outputs before and after injecting constant deviation fault are presented, respectively. (**a**) Derivative of ϕ; (**b**) Derivative of p; (**c**) Derivative of ψ; (**d**) Derivative of r.

For full validation, two constant deviation faults are injected on p, respectively, from the 87th to the 110th and the 347th to the 370th sample point. The amplitude of the injected fault is 2°. The results of the lateral outputs are given in Figure 6 and their values are transformed to degrees for illustrating. The faulty characteristics of the curves are intuitively shown in Figure 6. It can be seen that after constant deviation fault is injected to p, the other three parameters are also influenced. ϕ and ψ have presented obvious fault information with large variation in their values, while the change in r is relatively small, indicating that r is not quite sensitive to the change of p. It also can be seen that the sample points outside the fault injection intervals have also shown faulty characteristics, i.e., the values of the parameters require a transition stage to return back to the normal area. The samples before the faulty curve catches up with the normal curve are all regarded as faulty samples and adopted for testing the performance of fault detection approaches. Furthermore, the outliers are recognized by utilizing the 3σ criterion and expert knowledge, which is commonly adopted in practical engineering of UAV. Some approximation is made to simulate enough outliers. The derivatives of ϕ, ψ, p and r, are shown below in Figure 7, which are expected to improve the performance of the proposed approach because they can provide more spatial correlation information for the sensors according to the mechanism of the UAV. It can be seen that $\dot{\phi }$ and $\dot{\psi }$ are quite sensitive to constant deviation sensor fault, and have presented obvious fault information, while the change in $\dot{p}$ and $\dot{r}$ are not quite large. They are all adopted for supplementing the information to achieve higher fault detection performance.

**Figure 8:**
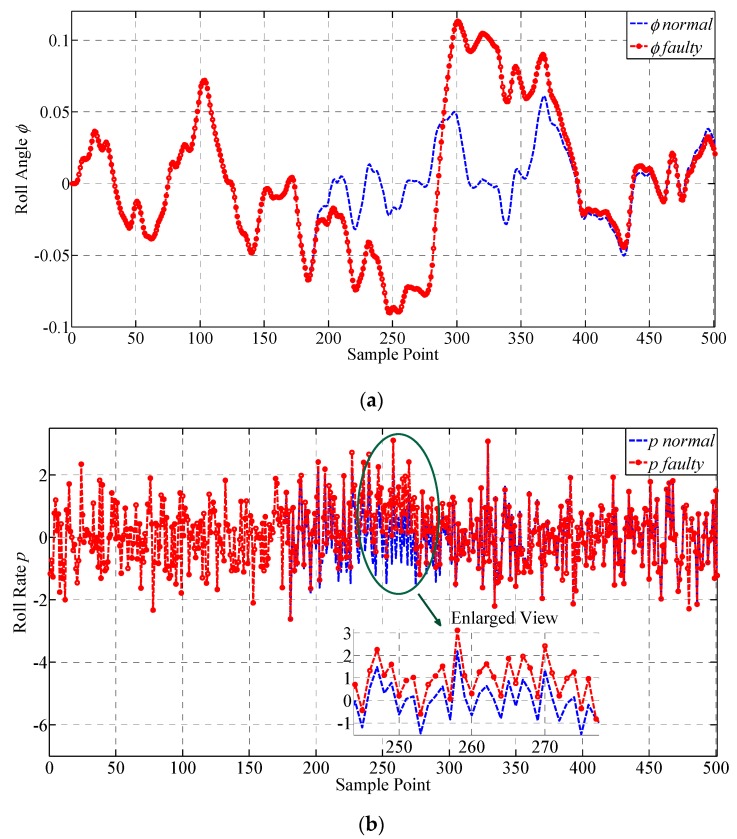

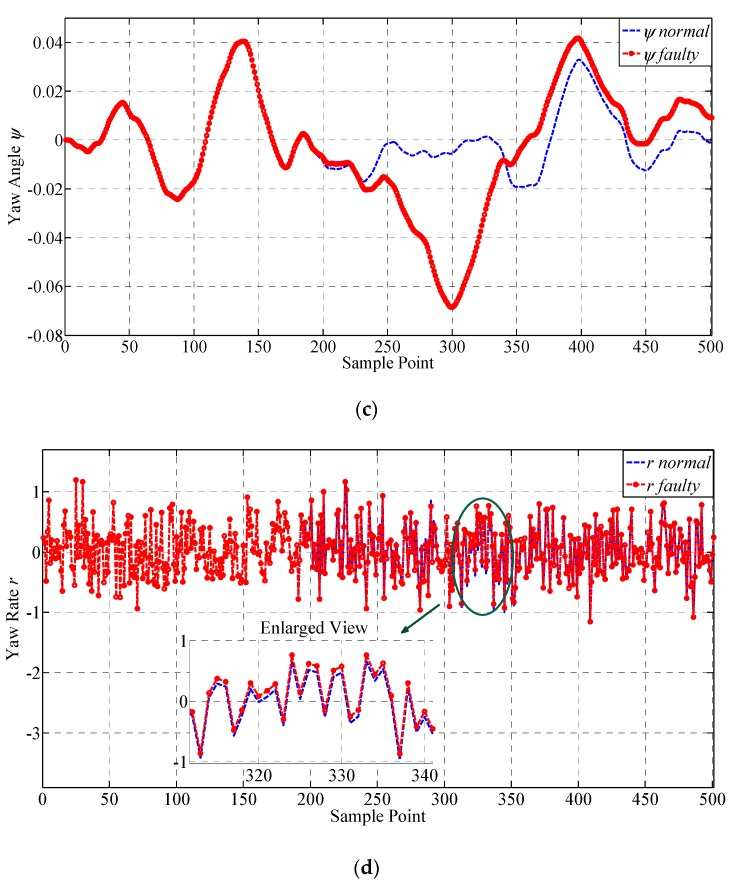
Illustration of the influence of drift fault on p to the lateral system. The curves of lateral outputs before and after injecting drift fault are presented, respectively. (**a**) Roll Angle ϕ; (**b**) Roll Rate p; (**c**) Yaw Angle ψ; (**d**) Yaw Rate r.

**Figure 9:**
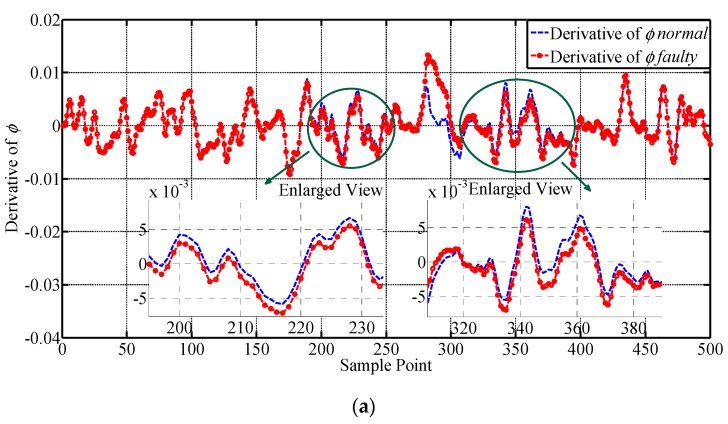

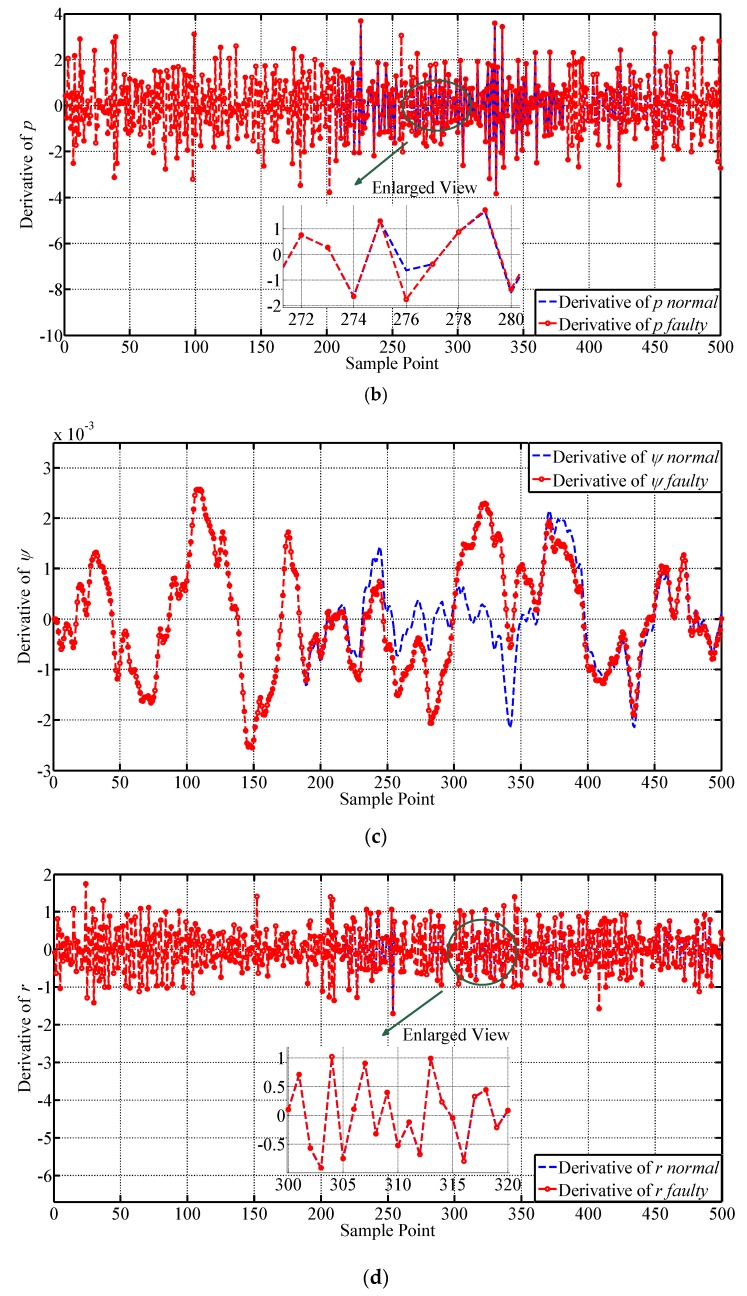
Illustration for the derivatives of the monitored lateral parameters. The curves of the derivatives of the lateral outputs before and after injecting drift fault are presented, respectively. (**a**) Derivative of ϕ; (**b**) Derivative of p; (**c**) Derivative of ψ; (**d**) Derivative of r.

A drift fault is injected on p from the 175th to the 275th sample point, with a step increase of 0.0115°. The results of the lateral outputs are given in Figure 8 and their values are transformed to degrees for illustrating. The faulty characteristics of the curves are intuitively shown by the figures in Figure 8. It can be seen that the drift fault injected does not have an obvious influence on the parameters until the 190th sample point, because the amplitude of the fault is quite small in the initial stage. The changes of the curves seem gentler comparing with those obtained under constant deviation fault. The value of the fault reaches its peak at the 275th sample point, and the following sample points have also exhibited faulty characteristics until the end of the process. ϕ and ψ have presented obvious faulty information while the change in r is relatively small, indicating that r is not quite sensitive to the change in p, which agrees with the experiment results under constant deviation fault. The samples are all regarded as faulty samples until the later outputs catch up with the normal outputs, which are then adopted for testing the performance of fault detection approaches. No additional outliers are identified. The derivatives of ϕ, ψ, p and r, are shown below in Figure 9, which are expected to improve the performance of the proposed approach because they can provide more spatial correlation information for the sensors according to the mechanism of the UAV. 

It can be seen that $\dot{\psi }$ is quite sensitive to drift sensor fault, and has presented obvious fault information from the 210th sample point. $\dot{\phi }$ and $\dot{p}$ have obvious change at the end of the drift fault, while the change in $\dot{r}$ is quite small. Comparing to constant deviation fault, the change in $\dot{\phi }$ and $\dot{p}$ are both weakened, because of different faulty characteristics. They are all adopted for supplementing the information to achieve higher fault detection performance.

### 3.2. Performance Validation Indices

Fault detection results can generally be divided into four different types, True Positive, False Positive, False Negative and True Negative, as shown in Table 3. To evaluate the fault detection performance of the fault detection approaches, True Positive rate (TPR) and False Positive Rate (FPR) are adopted in the experiments. Large TPR and small FPR values represent satisfactory detection performance of the detection approach. The definitions for TPR and FPR are given in Equations (17) and (18).

(17) $$\mathrm{TPR}=\frac{\mathrm{TP}}{\mathrm{TP}+\mathrm{FN}}$$

(18) $$\mathrm{FPR}=\frac{\mathrm{FP}}{\mathrm{TN}+\mathrm{FP}}$$

In addition, the Receiver Operating Characteristic (ROC) curve and the Area Under the Curve (AUC) value are also utilized for evaluating the performance of the proposed approach, which are evaluation indices generally adopted for fault detection. The ROC curve takes the FPR as the x-coordinate and the TPR as the y-coordinate, and can offer more performance information for the detection approaches with different pairs of TPRs and FPRs. The detection performance of the approaches with various detection thresholds can be compared using the ROC curves. The AUC values offer an overall performance evaluation of the detection approaches by computing their integral areas under the ROC curves.

### 3.3. Fault Detection Results of the Proposed Approach

**Figure 10:**
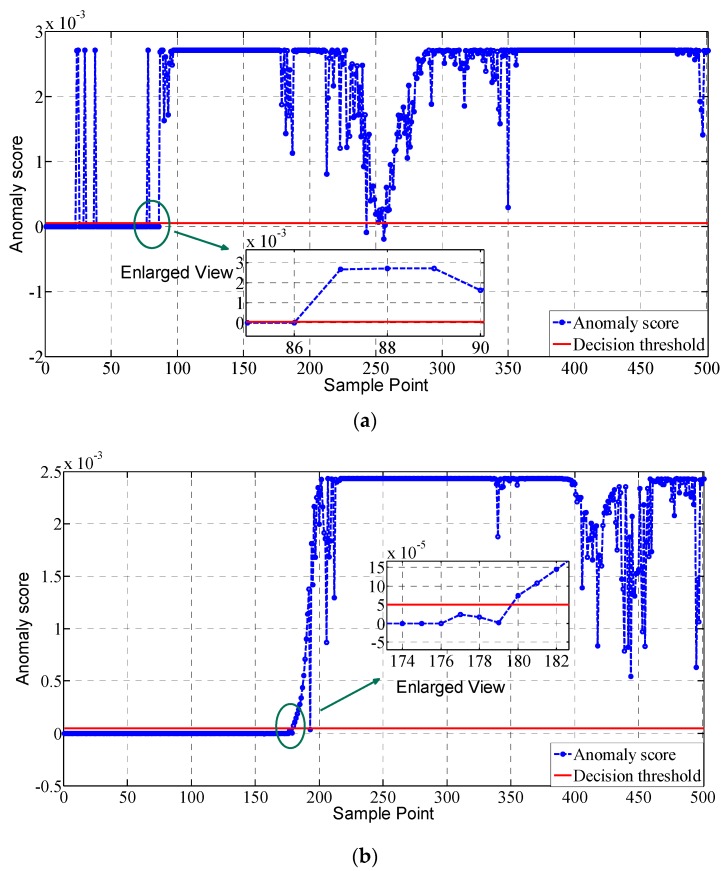
Fault detection results of the proposed approach under two different fault modes. (**a**) Constant deviation fault; (**b**) Drift fault.

The fault detection results adopting the proposed approach under two different fault modes are given in Figure 10. As can be seen from Figure 10, the constant deviation fault of the sensors can be detected immediately without time delay, which starts from the 87th sample point. In addition, the overall detection rate is quite satisfactory with the anomaly scores of the fault sample points far above the decision threshold and those of the normal points below. There exist some false alarms and missed detections, but the amount is small and acceptable. It can be concluded that through constructing the derivatives of the parameters and establishing the decision boundary adopting multiple dimensional variables, the detection performance of the proposed approach is quite satisfactory. Comparing to the constant deviation fault, the drift fault starts from the 175th sample point and is detected by the proposed approach from the 180th sample point, with a time delay of 5 sample points. This is because the influence of the drift fault is quite small at the initial stage and very difficult to be detected. With the increase in its fault amplitude, its fault feature becomes obvious and is then captured by the proposed approach. In addition, the detection rate of the proposed approach is also high in this fault mode with few false alarms and missed detections. We have also observed a decrease in anomaly score from the 210th sample point to the 280th sample point in the constant deviation fault mode and from the 400th sample point in the drift fault mode. This is because the fault extent is simulated to decrease and the flight control system will control the states to return back to the normal state. In conclusion, the proposed approach has presented favorable performance in both the two fault modes, with quick fault detection and high detection rate. Detailed detection performance indices of the proposed approach are compared with other typical approaches in the next section for further analysis and comparison.

Furthermore, the computational efficiency of the proposed approach is high because the detection model is just composed of some support vectors and only some simple multiplication operations are required to calculate the property of a testing sample. Therefore, the proposed approach is expected to be conducted online for real-time monitoring tasks. 

### 3.4. Comparison Results with Other Approaches

For illustrating the effectiveness of the proposed approach, comparison experiments with four other typical unsupervised fault detection approaches on two different types of sensor faults are conducted, using the flight sensor data acquired above. In addition to OCSVM, principle component analysis (PCA), kernel principle component analysis (KPCA) and local outlier factor (LOF) are also adopted for conducting comparison experiments, which are the representative of boundary-based, reconstruction-based and density-based approaches, respectively. The principle categories of unsupervised fault detection approaches are all contained to provide thorough comparison results for the proposed approach. 

The parameter v in OCSVM and the proposed approach is set to 0.01. The Gaussian kernel function is adopted in the proposed approach, which is the same for OCSVM and KPCA. The width parameter for the kernel function and the parameters μ and η in the proposed approach are all optimized through the gird search method. The confidence level for PCA and KPCA is set to 0.99, and the number of neighbors for LOF is 8. The variables are selected to achieve better performance.

**Figure 11:**
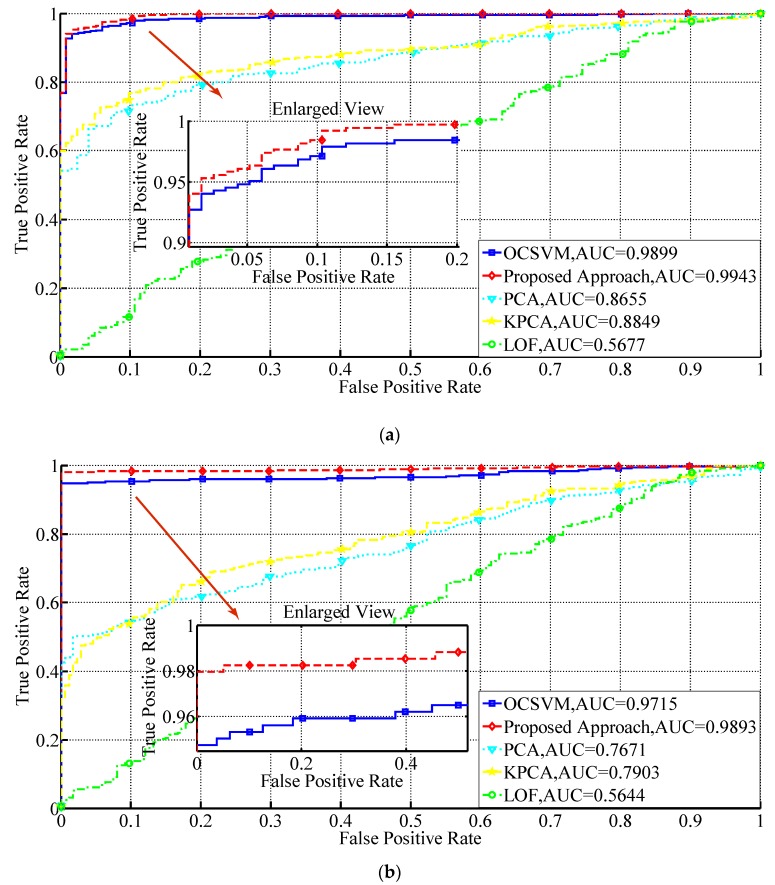
ROC curves for the proposed approach against other typical unsupervised fault detection approaches on two different types of sensor faults. (**a**) Constant deviation fault; (**b**) Drift fault.

The TPR, FPR and AUC values for the approaches under two different fault modes are given in Table 4 and the ROC curves for them are plotted in Figure 11. By comparing the results given in Table 4 and Figure 11, it can be seen that the proposed approach outperforms all the other approaches, with higher TPR, lower FPR and larger AUC values. It has obvious improvement in the three adopted performance indices in both the two fault modes comparing to OCSVM. The reason is that the influence of the outliers is mitigated by the proposed approach, and therefore, its decision boundary is more compact comparing with that of OCSVM, leading to an increase in detection accuracy. Also, the ROC curve of the proposed approach is always above that of OCSVM, indicating that it can obtain a higher positive detection rate with the same false alarm rate. The small decrease in detection performance of the proposed approach in the drift fault mode is because the faulty instances in this mode are small in amplitude in the initial stage and hard to detect. The problem is worse for the other approaches. Overall, the two algorithms have both achieved satisfactory performance on the two datasets, with low false alarm rates in the initial stage of the ROC curves.

The AUC values for PCA and KPCA on constant deviation fault are 0.8655 and 0.8849, respectively. However, they decrease to 0.7671 and 0.7903 in the case of drift fault, which indicates that PCA and KPCA have worse performance on detecting faults with small amplitude. This is because the influence of drift fault on the parameters is quite small in the initial stage and it requires the algorithms to be capable of detecting small faults. The decreases are much larger than those of the proposed approach and OCSVM, indicating that boundary based approaches can achieve better performance in detecting small faults. Besides, KPCA has achieved better performance comparing to PCA with its kernel parameter optimized through the grid search method, because the nonlinear characteristics of the sensor data are more properly dealt with. The outliers may have also influenced the detection performance in the constant deviation fault condition. For the LOF approach, its performance is not quite satisfactory in both the two cases, with its AUC values being 0.5677 and 0.5644, respectively, which is because LOF is intended primarily for detecting scattered fault samples and consecutive fault instances with high local densities will be judged as normal, leading to a high FPR. For continuous faulty instances, LOF is not quite appropriate.

In conclusion, the proposed approach has achieved the best detection performance among all the approaches by comparing the FPR, TPR and AUC indices with other approaches, which has illustrated its superior capability of detecting small faults and dealing with the outliers. Also, boundary based algorithms, represented by the proposed approach and OCSVM, have outperformed the reconstruction based algorithms, represented by PCA and KPCA, and the density based approach represented by LOF in both the two fault cases. The experimental results have emphasized the effectiveness of the proposed approach.

## 4. Conclusions

In this paper, an unmanned aerial vehicle sensor fault detection approach using classifier without negative samples is proposed, which can be seen as a local density regulated optimization in one-class support vector machine approach. The proposed approach can handle the effect of outliers effectively and obtain a compact boundary for detecting flight sensor faults with small amplitude. By regulating the coefficients of the outliers according to their local densities through a well-established rule, the influence of them is mitigated in the proposed approach. Also, by narrowing the application scope of the local density theory, the basic structure of one-class support vector machine is reserved and unnecessary computation is avoided. Comparison experiments with other four approaches belonging to three different categories on two different types of common sensor faults for the unmanned aerial vehicle are conducted. Three performance indices are adopted to thoroughly evaluate the performance of the proposed approach and the detection results have proved its effectiveness. In the future, we plan to investigate the online update strategy for the proposed approach to extend its application area. In addition, we will validate the performance of our approach on real unmanned aerial vehicle fault data to improve its creditability.

## Figures and Tables

**Table 1:** Parameters for the UAV simulation model.

Parameter	Definition	Unit
ϕ	Roll Angle	rad
θ	Pitch Angle	rad
ψ	Yaw Angle	rad
p	Roll Rate	rad/s
q	Pitch Rate	rad/s
r	Yaw Rate	rad/s
$a_{x}$	X-axis Acceleration	m/s^2^
$a_{y}$	Y-axis Acceleration	m/s^2^
$a_{z}$	Z-axis Acceleration	m/s^2^
$v_{s}$	Air Speed	m/s
α	Angle of Attack	rad
β	Angle of Sideslip	rad
h	Altitude	m

**Table 2:** Parameters for monitoring the lateral subsystem.

Parameter	Definition	Unit
ϕ	Roll Angle	rad
ψ	Yaw Angle	rad
p	Roll Rate	rad/s
r	Yaw Rate	rad/s
$\dot{\phi }$	Derivative of Roll Angle	rad/s
$\dot{\psi }$	Derivative of Yaw Angle	rad/s
$\dot{p}$	Derivative of Roll Rate	rad/s^2^
$\dot{r}$	Derivative of Yaw Rate	rad/s^2^

**Table 3:** The types of detection results.

Detection Results	Normal Data	Faulty Data
Normal	True Positive (TP)	False Positive (FP)
Faulty	False Negative (FN)	True Negative (TN)

**Table 4:** Performance indices for the proposed approach against other typical fault detection approaches on two different types of sensor faults.

Fault Type	Performance Index	Proposed Approach	OCSVM	PCA	KPCA	LOF
Constant deviation fault	AUC	0.9943	0.9899	0.8655	0.8849	0.5677
	TPR	0.9629	0.9526	0.8725	0.8840	0.9406
	FPR	0.0508	0.0539	0.4734	0.5266	0.8533
Drift fault	AUC	0.9893	0.9715	0.7671	0.7903	0.5644
	TPR	0.9796	0.9512	0.7278	0.8353	0.8678
	FPR	0.0404	0.0525	0.4006	0.5321	0.8073

## References

[1] Wang B., Liu D., Wang W., Peng X. (2018). A hybrid approach for UAV flight data estimation and prediction based on flight mode recognition. Microelectron. Reliab..

[2] Avram R.C., Zhang X., Campbell J., Muse J. (2015). IMU sensor fault diagnosis and estimation for quadrotor UAVs. IFAC-PapersOnLine.

[3] Khalastchi E., Kalech M., Kaminka G.A., Lin R. (2015). Online data-driven anomaly detection in autonomous robots. Knowl. Inf. Syst..

[4] Suarez A., Heredia G., Ollero A. (2018). Cooperative Virtual Sensor for Fault Detection and Identification in Multi-UAV Applications. J. Sens..

[5] Wang L., Li M., Liu L., Liu D. Exhaust gas temperature sensing data anomaly detection for aircraft auxiliary power unit condition monitoring. Proceedings of the 2018 International Conference on Sensing, Diagnostics, Prognostics, and Control (SDPC).

[6] Gao Z., Cecati C., Ding S.X. (2015). A survey of fault diagnosis and fault-tolerant techniques Part I: Fault diagnosis. IEEE Trans. Ind. Electron..

[7] Ortiz-Torres G., López-Estrada F.R., Reyes-Reyes J., García-Beltrán C.D., Theilliol D. (2016). An Actuator Fault Detection and Isolation method design for Planar Vertical Take-off and Landing Unmanned Aerial Vehicle modelled as a qLPV system. IFAC-PapersOnLine.

[8] Abbaspour A., Aboutalebi P., Yen K.K., Sargolzaei A. (2017). Neural adaptive observer-based sensor and actuator fault detection in nonlinear systems: Application in UAV. ISA Trans..

[9] López-Estrada F.R., Ponsart J.C., Theilliol D., Zhang Y., Astorga-Zaragoza C.M. (2016). LPV Model-Based Tracking Control and Robust Sensor Fault Diagnosis for a Quadrotor UAV. J. Intell. Robot. Syst. Theory Appl..

[10] Liu D., Yin X., Song Y., Liu W., Peng Y. (2018). An on-line state of health estimation of lithium-ion battery using unscented particle filter. IEEE Access.

[11] Zhang Y., Liu L., Peng Y., Liu D. (2018). An Electro-Mechanical Actuator motor voltage estimation method with a feature-aided Kalman Filter. Sensors.

[12] Liu D., Song Y., Li L., Liao H., Peng Y. (2018). On-line life cycle health assessment for lithium-ion battery in electric vehicles. J. Clean. Prod..

[13] Liu L., Wang S., Liu D., Peng Y. (2017). Quantitative selection of sensor data based on improved permutation entropy for system remaining useful life prediction. Microelectron. Reliab..

[14] Zhao G., Liu X., Zhang B., Liu Y., Niu G., Hu C. (2018). A novel approach for analog circuit fault diagnosis based on deep belief network. Measurement.

[15] Zhang S., He Q., Ouyang K., Xiong W. (2018). Multi-bearing weak defect detection for wayside acoustic diagnosis based on a time-varying spatial filtering rearrangement. Mech. Syst. Signal Process..

[16] Zhao R., Wang D., Yan R., Mao K., Shen F., Wang J. (2018). Machine Health Monitoring Using Local Feature-Based Gated Recurrent Unit Networks. IEEE Trans. Ind. Electron..

[17] Wang D., Miao Q. (2015). Smoothness index-guided Bayesian inference for determining joint posterior probability distributions of anti-symmetric real Laplace wavelet parameters for identification of different bearing faults. J. Sound Vib..

[18] Zhang W., Du L., Li L., Zhang X., Liu H. (2018). Infinite Bayesian one-class support vector machine based on Dirichlet process mixture clustering. Pattern Recognit..

[19] Zhang Y., Du B., Zhang L., Wang S. (2016). A low-rank and sparse matrix decomposition-based mahalanobis distance method for hyperspectral anomaly detection. IEEE Trans. Geosci. Remote Sens..

[20] Yuan J., Yuan R., Chen X. (2014). Network Anomaly Detection Based on Multi-scale Dynamic Characteristics of Traffic. Int. J. Comput. Commun. Control.

[21] Kittler J., Christmas W., De Campos T., Windridge D., Yan F., Illingworth J., Osman M. (2014). Domain anomaly detection in machine perception: A system architecture and taxonomy. IEEE Trans. Pattern Anal. Mach. Intell..

[22] Jiang Q., Yan X. (2015). Nonlinear plant-wide process monitoring using MI-spectral clustering and Bayesian inference-based multiblock KPCA. J. Process Control.

[23] Dorobantu A., Ozdemir A.A., Turkoglu K., Freeman P., Murch A., Mettler B., Balas G. Frequency Domain System Identification for a Small, Low-Cost, Fixed-Wing UAV. Proceedings of the AIAA Guidance, Navigation, and Control Conference.

[24] Goodrich M.A., Morse B.S., Gerhardt D., Cooper J.L., Quigley M., Adams J.A., Humphrey C. (2008). Supporting wilderness search and rescue using a camera-equipped mini UAV. J. F. Robot..

[25] Liu Y., Bazzi A.M. (2017). A review and comparison of fault detection and diagnosis methods for squirrel-cage induction motors: State of the art. ISA Trans..

[26] Ilonen J., Paalanen P., Kamarainen J.-K., Kälviäinen H. Gaussian mixture pdf in one-class classification: Computing and utilizing confidence values. Proceedings of the 18th International Conference on Pattern Recognition.

[27] Song B., Shi H., Ma Y., Wang J. (2014). Multisubspace principal component analysis with local outlier factor for multimode process monitoring. Ind. Eng. Chem. Res..

[28] Capozzoli A., Lauro F., Khan I. (2015). Fault detection analysis using data mining techniques for a cluster of smart office buildings. Expert Syst. Appl..

[29] Zhou F., Park J.H., Liu Y. (2016). Differential feature based hierarchical PCA fault detection method for dynamic fault. Neurocomputing.

[30] Yan K., Ji Z., Shen W. (2017). Online fault detection methods for chillers combining extended kalman filter and recursive one-class SVM. Neurocomputing.

[31] Fu C., Duan R., Kircali D., Kayacan E. (2016). Onboard robust visual tracking for UAVs using a reliable global-local object model. Sensors.

[32] Jwa S., Özgüner Ü., Tang Z. (2008). Information-theoretic data registration for UAV-based sensing. IEEE Trans. Intell. Transp. Syst..

[33] Xiao Y., Wang H., Xu W., Zhou J. (2016). Robust one-class SVM for fault detection. Chemom. Intell. Lab. Syst..

[34] Amer M., Goldstein M., Abdennadher S. Enhancing one-class support vector machines for unsupervised anomaly detection. Proceedings of the ACM SIGKDD Workshop on Outlier Detection and Description.

[35] Erfani S.M., Rajasegarar S., Karunasekera S., Leckie C. (2016). High-dimensional and large-scale anomaly detection using a linear one-class SVM with deep learning. Pattern Recognit..

[36] Aboutalebi P., Abbaspour A., Forouzannezhad P., Sargolzaei A. (2018). A Novel Sensor Fault Detection in an Unmanned Quadrotor Based on Adaptive Neural Observer. J. Intell. Robot. Syst..

[37] Sun R., Cheng Q., Wang G., Ochieng W.Y. (2017). A Novel Online Data-Driven Algorithm for Detecting UAV Navigation Sensor Faults. Sensors.

[38] Caliskan F., Hajiyev C. (2016). Active Fault-Tolerant Control of UAV Dynamics against Sensor-Actuator Failures. J. Aerosp. Eng..

[39] Paw Y.C. (2009). Synthesis and validation of flight control for UAV. Ph.D. Thesis.

[40] Freeman P., Pandita R., Srivastava N., Balas G.J. (2013). Model-Based and Data-Driven Fault Detection Performance for a Small UAV. IEEE Trans. Mechatron..

